# A Shift from Standard Median Sternotomy to Robotic-Assisted Thoracic Surgery for Resection of Anterior Mediastinal Tumors

**DOI:** 10.3390/jcm15020638

**Published:** 2026-01-13

**Authors:** Michael Peer, Sharbel Azzam, Nachum Nesher, Marina Kolodii, Yaacov Abramov, Vladimir Verenkin, Ruth Shaylor, Arnon Karni, Avi Gadoth, Eugenio Pompeo, Idit Matot, Ofer Merimsky

**Affiliations:** 1 Department of Thoracic Surgery, Tel Aviv Medical Center, Faculty of Medicine, Tel Aviv University, Weitzman Street 6, Tel Aviv 6423906, Israel; sharbelazzam8@hotmail.com (S.A.); nesher61@gmail.com (N.N.); marina05060@gmail.com (M.K.); yyakov@msn.com (Y.A.); 2 Division of Anesthesiology, Intensive Care and Pain, Tel Aviv Medical Center, Faculty of Medicine, Tel Aviv University, Weitzman Street 6, Tel Aviv 6423906, Israel; vladimirv@tlvmc.gov.il (V.V.); ruth@shaylor.co.uk (R.S.); iditm@tlvmc.gov.il (I.M.); 3 Neurology Institute, Tel Aviv Medical Center, Faculty of Medicine, Tel Aviv University, Weitzman Street 6, Tel Aviv 6423906, Israel; arnonk@tlvmc.gov.il (A.K.); avig@tlvmc.gov.il (A.G.); 4 Department of Thoracic Surgery, Tor Vergata University of Rome, 00133 Rome, Italy; pompeo@med.uniroma2.it; 5 Division of Oncology, Tel Aviv Medical Center, Faculty of Medicine, Tel Aviv University, Weitzman Street 6, Tel Aviv 6423906, Israel; oferm@tlvmc.gov.il

**Keywords:** median sternotomy, RATS (robotic-assisted thoracic surgery), thymectomy

## Abstract

**Objectives:** Robotic-Assisted Thoracic Surgery (RATS) has emerged as a viable alternative to traditional median sternotomy for patients with anterior mediastinal tumors suspected of having thymoma or those with Myasthenia Gravis (MG). While median sternotomy remains a widely accepted standard approach, RATS has gained popularity due to its potential benefits. **Methods:** We retrospectively reviewed our 5 years’ experience of performing 111 surgeries for patients with anterior mediastinal tumors and patients with MG suspected of having thymoma. We performed multivariate regression models to assess the association between main demographic and clinical variables and two primary outcomes: overall complications and hospital stay. **Results:** Out of 111 patients, 54 were men (48.6%) and 57 were women (51.4%). The majority of surgeries (n = 93) were performed by RATS (83.8%), while the remainder were performed by either median sternotomy (n = 15, 13.5%) or by other approaches (n = 3, 2.7%). Sixty-five patients were diagnosed with thymoma (58.6%), with 96.9% R0 resection. Sixty-five patients underwent left-sided surgery (58.6%), and thirty-one underwent right-sided surgery (27.9%). The conversion rate was 2.5%. The rate of postoperative complications was 8.1 without perioperative mortality. The median hospital stay was 4.62 days, but it was significantly shorter in the RATS compared to the median sternotomy group (mean 3.64 vs. 10.67 days, *p* = 0.040). **Conclusions:** Our results suggest that RATS for patients with anterior mediastinal tumors suspected of having thymoma or for those with MG is safe and technically feasible and may be the preferred surgical approach for selected patients, whereas traditional median sternotomy remains the preferred choice for more locally advanced tumors.

## 1. Introduction

Thymomas are the most common primary tumors in the anterior mediastinum, characterized by slow growth, occasional local invasion, intrathoracic spread, and rare distant metastases. Median sternotomy followed by complete resection of the tumor together with mediastinal fat with cervical and pericardial horns of the thymus is considered the standard surgical approach for thymectomy. The goal of an appropriate surgical approach is to provide excellent access to the anterior mediastinum and enable technically safe surgery. Today, minimally invasive surgical approaches such as Video-Assisted Thoracic Surgery (VATS) and Robotic-Assisted Thoracic Surgery (RATS) have emerged as alternatives to open surgery. VATS, in comparison to traditional median sternotomy, prefers a two-dimensional platform for surgery, and RATS prefers a three-dimensional platform. Two meta-analyses [1,2] that compared RATS and VATS thymectomies concluded that RATS is superior to VATS because it is associated with decreased intraoperative blood loss, shorter drainage time, lower rate of postoperative complications, and shorter hospital stay. A more recent meta-analysis [3] showed similar advantages for RATS in comparison with median sternotomy.

The present study aims to analyze a five-year experience in performing anterior mediastinal tumor surgical resection at the Department of Thoracic Surgery, Tel Aviv Medical Center, and to compare clinical and surgical outcomes between RATS and median sternotomy in order to assess the potential advantages, limitations, and safety profile of RATS.

## 2. Materials and Methods

### 2.1. Patient Population

We retrospectively reviewed our 5 years’ experience in performing surgeries for patients with anterior mediastinal tumors and patients with MG suspected of having thymoma. Data were retrieved from the electronic medical records and included preoperative, intraoperative, and postoperative characteristics of the patients, including conversion rate, complications, histopathologic analysis of the tumors, stage stratification for MG patients, hospital stay, adjuvant radiotherapy or chemotherapy, tumor recurrence, and survival. Follow-up data for all the patients were updated to 1 January 2025. The article contains only anonymous and unidentifiable data.

### 2.2. Inclusion and Exclusion Criteria

Surgery was planned when preoperative computed tomography (CT) of the chest demonstrated an anterior mediastinal tumor with features highly suspected of having thymoma, or in patients with MG. Inclusion criteria for choosing the RATS approach, as suggested by Cheng et al. [4], were an encapsulated tumor in the anterior mediastinum without signs of invasion of surrounding great vessels, including a clear fat plane between the tumor and mediastinal structures, and unilateral tumor predominance. The side of RATS was decided according to the laterality of the tumor in the anterior mediastinum. Exclusion criteria for RATS were a tumor in the anterior mediastinum without a clear fat plane between the tumor and mediastinal structures, or the presence of signs of vascular compression, or a tumor crossing the midline in the anterior mediastinum. The patients with MG without a distinct tumor presentation in the anterior mediastinum were also planned for RATS, generally by a left-sided approach. The size of the tumor was not a contraindication for RATS, except in cases of tumors with bilateral to midline localization in the anterior mediastinum.

### 2.3. Perioperative Period

Preoperative evaluation included blood count, chemistry, and coagulation tests, electrocardiography, chest radiography, pulmonary function tests (PFT), and CT of the chest with contrast. Magnetic Resonance Imaging (MRI) of the chest was performed in selected patients.

Surgery in all cases included en bloc thymectomy with resection of all mediastinal fat and cervical and pericardial horns of the thymus. The resection borders were the lower pericardium caudally, the innominate vein cranially, and the phrenic nerves bilaterally.

Median sternotomy was performed by standard technique with the patient in the supine position with a roll placed under the shoulders to expose the anterior mediastinum. RATS was performed by the Da Vinci Xi Surgical System (Intuitive Surgical Inc., Sunnyvale, CA, USA) through a three-arm unilateral robotic approach [5]. The patients were placed left-side or right-side up to a 15–30 angle according to the side of the procedure, and for better visualization of the contralateral phrenic nerve. The ipsilateral arm was positioned with partial extension to avoid damage to the brachial plexus, and the chest was prepared for possible median sternotomy in all cases. The first 12 mm camera incision was performed at the fifth intercostal space in the anterior axillary line, and carbon dioxide (CO_2_) was insufflated into the chest (up to a pressure of 8 to 10 mmHg) to enlarge the operating space, and the robotic camera with a 3-dimensional 30° stereo endoscope was inserted. Second 8 mm port incision, at the third intercostal space, and third 8 mm port incision, at the eighth intercostal space, in the mid-axillary and mid-clavicular lines, respectively, were made for two atraumatic robotic instruments: the right handheld Maryland bipolar forceps and the left handheld Cadiere forceps (Endo-Wrist; Intuitive Surgical, Inc., Sunnyvale, CA, USA) [5].

Standardized robotic ports targeted far from the mediastinal tumor maximally enlarged operative space that, together with CO_2_ insufflation, enabled us to perform a safe dissection for larger mediastinal tumors. The tumor with the mediastinal fat and four thymic horns was removed en bloc by the Endo Catch 10 (Medtronic Intermodal Services, Inc., Cincinnati, OH, USA) bag by widening the third robotic port incision at the eighth intercostal space in the mid-clavicular line up to 5–6 cm along the intercostal space, which allows extraction of even larger tumors.

Airway management for RATS included a double-lumen endotracheal tube for split-lung ventilation or a bronchial blocker, generally for left-side surgery, while for median sternotomy, a single-lumen endotracheal tube was placed. Intraoperative monitoring included blood pressure monitors (invasive and noninvasive), ECG, pulse oximetry, body temperature, and neuromuscular blockage monitoring. Thoracic epidural anesthesia (TEA) or paravertebral block (PVB) was used according to our preoperative local infiltration anesthesia (LIA) protocol.

### 2.4. Histopathology

The Masaoka staging system was used for pathologic staging of thymoma [6]. The New World Health Organization Histologic Classification System was used for the definition of thymoma histology [7]. MG is classified by the Myasthenia Gravis Foundation of America (MGFA) grading system for severity (Class I: ocular only; Class II: mild generalized; Class III: moderate; Class IV: severe; Class V: intubation/ventilation) [8]. R0 resection was defined as the absence of the tumor (thymoma) on the surgical margins (including microscopically invasive tumor (thymoma) capsular or completely resected pericardium or adjacent structures (phrenic nerves or lungs). R1 resection was defined as the permanence of microscopic residual tumor (thymoma) cells in the thymic or mediastinal fat [9].

All patients with thymomas or other malignancies were referred to the thoracic oncology service, and patients with MG to the neurology service. Post-operative radiation therapy was delivered to all patients with invasion of the capsular or mediastinal fat. Follow-up by CT with contrast was performed every 6 months for the first two years and once a year thereafter.

### 2.5. Data Analysis

Data was analyzed using SPSS version 28.0. First, we produced descriptive statistics for all variables, presenting frequencies with absolute numbers (N) and percentages for discrete categorical variables (e.g., sex) and means with standard deviations for continuous variables (e.g., age). We compared patients undergoing RATS and those treated via median sternotomy by using chi-square tests for discrete categorical variables and independent *t*-tests for continuous variables. Finally, we performed multivariate regression models to assess the association between main demographic and clinical variables and two main outcomes—overall complications (logistic regression) and hospital stay (linear regression). For each variable, the model presents coefficients (odds ratio (OR) for logistic regression and B for linear regression), *p*-value, and 95% confidence intervals.

Given the substantial imbalance in group sizes and baseline age between surgical approaches, multivariable regression models were used to adjust for age and other clinically relevant covariates when examining hospital length of stay. These analyses were considered exploratory and intended to account for measured confounding rather than establish causal inference.

A *p*-value lower than 5% was considered a significant result.

## 3. Results

### 3.1. Demographic and Clinical Descriptive Statistics of the Cohort

One hundred eleven surgeries for anterior mediastinal tumors highly suspected of having thymoma, with or without MG, and patients with MG were performed at our institution from March 2019 to April 2024.

Of 111 patients, 54 were men (48.6%) and 57 were women (51.4%), with a mean age of 57.2 (range, from 9 to 88 years). The majority of surgeries (n = 93, 83.8%) were performed by RATS, while the remainder were by either median sternotomy (n = 15, 13.5%), VATS (n = 2, 2.5%), or thoracotomy (n = 1, 1.2%). Sixty-five (58.6%) patients were primarily diagnosed with thymoma, and 38 (34.2%) patients had MG, of whom 17 were found to have thymoma (15.3%).

Out of the 65 patients who underwent left-sided procedures, there were 63 RATS, one VATS, and one thoracotomy. Out of the 31 patients who underwent right-sided procedures, there were 30 RATS and one VATS. Out of 38 patients with MG, 7 patients were classified with Class I MG (18.4%), 16 with Class II (42.1%), 11 with Class III (28.9%), 3 with Class IV (7.9%), and 1 patient with Class V MG (2.6%).

### 3.2. Perioperative Morbidity and Mortality

All patients were extubated at the end of surgery. The mean hospital stay was 4.62 days (range, from two to 60 days). There was no perioperative mortality; the conversion rate from RATS to median sternotomy was 2.5% (two patients). Thirteen postoperative complications occurred in nine patients (8.1%). Atrial fibrillation occurred in three patients, intraoperative bleeding requiring conversion to median sternotomy in one patient, postoperative clotted hemothorax requiring VATS in one patient, phrenic nerve palsy in two patients, empyema treated by 14 French pigtail chest tube and fibrinolysis in two patients, and recurrent laryngeal nerve paresis in one patient. One patient with MG developed myasthenic crisis, bilateral pneumonia, prolonged mechanical ventilation, and tracheostomy (Table 1). The median of the follow-up period was 11.0 months.

### 3.3. Histopathology and Additional Treatment

**Figure 1:**
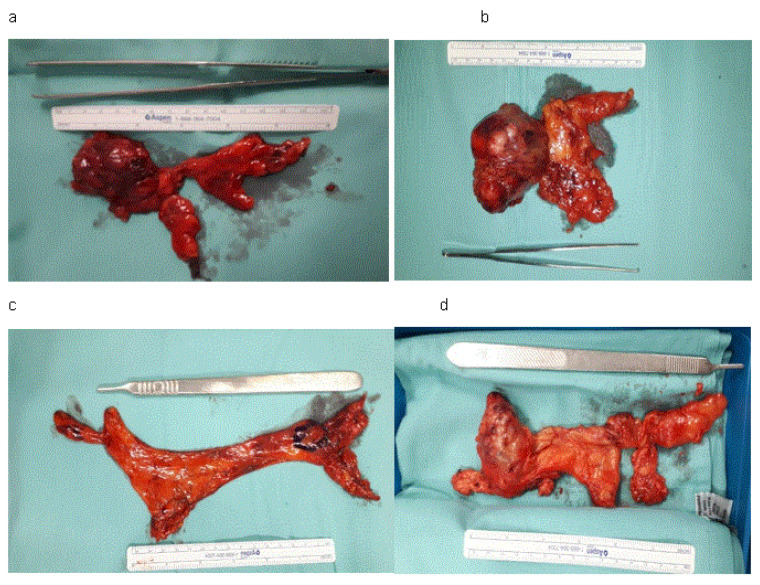
(**a**–**d**). Four patients after RATS en bloc thymectomies. (**a**) The patient after RATS thymectomy. (**b**) The patient after RATS thymectomy. (**c**) The patient after RATS thymectomy. (**d**) The patient after RATS thymectomy.

Sixty-five patients diagnosed with thymoma (mean size of 6.9 cm, range from 1.5 cm to 12.0 cm), 29 of them (44.6%) were diagnosed with AB thymoma, 21 (32.3%) with A thymoma, 8 (12.3%) with B1 thymoma, 4 (6.2%) with B2 thymoma, and 3 (4.6%) with B3 thymoma (Figure 1a–d).

**Figure 2:**
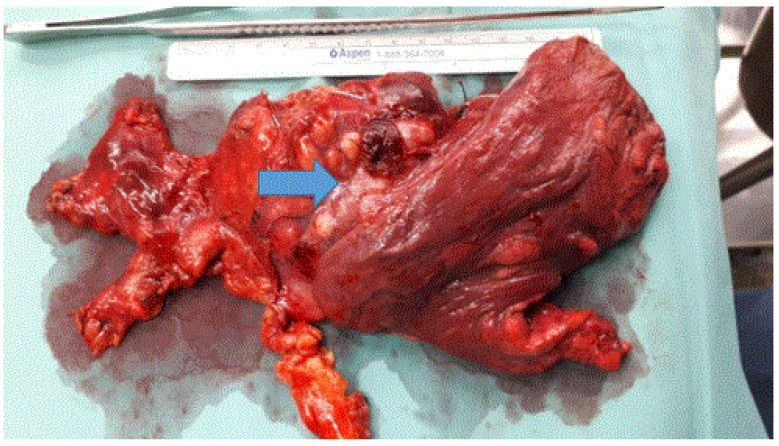
Resected thymoma and mediastinal fat involving the left upper lobe. Patient after staged median sternotomy and left posterolateral thoracotomy with en bloc thymectomy, left upper lobe lobectomy, and total pericardiectomy (the arrow: the completely resected thymoma and mediastinal fat involving the left upper lobe).

Sixty-three out of sixty-five patients diagnosed with thymomas had R0 resection (96.9%) (Table 1). Seven patients underwent pericardial resection due to direct pericardial involvement by the tumor, three of them operated on by RATS. One patient converted to median sternotomy due to intraoperative bleeding, one to left anterior thoracotomy due to pulmonary involvement, and two were initially performed by median sternotomy with one of them being staged as left posterolateral thoracotomy with left upper lobectomy and complete pericardial resection and reconstruction by bovine pericardium (Gore-Tex, cardiovascular patch, W.L. Gore & Associates, Inc., Newark, DE, USA) (Figure 2).

Seven patients were diagnosed with microscopic invasion of the tumor capsule, two of them with microscopic residual tumor cells in the thymic or mediastinal fat, and four with direct involvement and completely resected surrounding structures (phrenic nerves and lungs).

Postoperative radiation therapy was delivered to six patients with microscopically invasive tumors (thymoma) capsular (two of them with thymic and mediastinal fat invasion). One patient refused the treatment. Of seven patients with pericardial resection, only one was treated with adjuvant radiotherapy due to concurrent capsular invasion, and one with adjuvant chemotherapy due to diaphragmatic and pleural metastases.

Of 38 patients operated on due to presumed MG, 17 were diagnosed with thymoma, 20 with thymic hyperplasia, and one with thymic cyst. Of the remaining 25 patients, 6 were diagnosed with thymic hyperplasia, 5 with bronchogenic cyst, 2 with thymic carcinoma, 3 with thymic cyst, 6 with teratoma, paraganglioma, carcinoid tumor, lymphoma, cavernous hemangioma, and retrosternal goiter, respectively, and 3 with other benign thymic pathology (Table 2).

All patients diagnosed with thymoma are alive to date; one patient with AB thymoma was diagnosed with unilateral pleural metastasis 2 years after RATS and treated by total pleurectomy with Hyperthermic Intrathoracic Chemotherapy (HITHOC). Two patients diagnosed with thymic carcinoma postoperatively were treated by chemotherapy; one of them died two years after the surgery, and the second is alive.

### 3.4. RATS vs. Median Sternotomy

The comparison between RATS (n = 93) via median sternotomy (n = 15) in the main study variables revealed several notable differences (Table 3).

There were no significant differences in sex distribution between the groups (χ^2^ = 0.46, *p* = 0.49), nor in the prevalence of chronic diseases (66.7% in the sternotomy group vs. 63.4% in the RATS group; χ^2^ = 0.06, *p* = 0.81). The proportion of patients was higher in the sternotomy group (100.0%) compared to the RATS group (80.6%), though this difference did not reach statistical significance (χ^2^ =3.48, *p* = 0.070). Patients after RATS were significantly younger than those undergoing sternotomy (mean age 53.37 vs. 73.07 years, t =2.35, *p* = 0.002). No significant difference was found in postoperative complication rates between groups (6.7% vs. 9.7%; χ^2^ = 0.14, *p* = 0.709).

### 3.5. Multivariate Models Predicting Overall Complications and Hospital Stay

After adjusting for potential confounders by logistic regression (for overall complications) and linear regression (for hospital stay), no significant difference was found between the RATS vs. sternotomy groups (OR = 2.19, *p* = 0.56) (Table 4), but the average hospital stay was significantly lower among patients who underwent RATS in comparison with median sternotomy (mean 3.64 vs. 10.67 days; t = 1.88, *p* = 0.040); (B = −0.46, *p* < 0.001) (Table 5).

## 4. Discussion

The robotic thoracic surgery program at our hospital was initiated in April 2019, immediately after the foundation of the Thoracic Surgery Department earlier in 2019. Our RATS thymectomies program was supervised by the department director without previous experience in other minimally invasive techniques, such as VATS, subxiphoid, transcervical thymectomies, or others [5]. Actually, the present retrospective study on the surgical approach for anterior mediastinal tumors points to the successful selection of the surgical approach, whether RATS or median sternotomy. The data presented in this study underline the feasibility, efficacy, and safety of the RATS approach, as reflected by the high rate of R0 resection, low incidence of perioperative complications, and acceptable short-term oncologic outcome.

Our series results show that the evolution of RATS allowed us to operate on locally advanced anterior mediastinal tumors by this surgical approach, especially thymomas, with no inferiority when compared to median sternotomy. Careful patient selection according to radiologic findings preoperatively is mandatory in order to choose the appropriate surgical approach and maintain a low intraoperative conversion rate.

Thymectomy technique remains the main strategy of treatment of anterior mediastinal tumors suspected of having thymoma. Over the past decade, RATS has been increasingly used to perform thymectomy. The advantages of RATS are considerable, generally due to articulation with 7 degrees of freedom of robotic arms and 360 degrees of flexibility, which are well-suited for operating in narrow spaces such as the mediastinum. RATS is associated with less operative blood loss, shorter drainage time, fewer postoperative complications, potentially shorter hospital stays, and lower conversion rate to open surgery [1,2,3]. RATS has also been shown to be safe and technically feasible for 4–5 cm thymomas [10,11,12], and, recently, for larger 9.5–10 cm thymomas [13]. The mean size of thymomas in our study was 6.3 cm. Regarding the RATS for MG patients, without a distinct tumor presentation in the anterior mediastinum, the left-sided approach is preferred by us over a right-sided approach [14].

An additional point addressed in our study is R resection (margin status). Earlier, Weis et al. reported that margin status after thymectomy closely correlates with the risk of tumor recurrence [15]. Therefore, avoiding an R1 resection is the most important goal during RATS. Theoretically, RATS for large thymomas have a higher risk of capsular injury. For example, Kimura et al. [16] demonstrated that the VATS approach for thymectomy in tumors larger than 5 cm substantially increases the risk of capsular injury. Nachira et al. [17] showed the surgical and oncological effectiveness of the RATS thymectomy for large thymomas up to 12 cm in terms of radicality, complications, and in-hospital stay.

In our study, patients with microscopically invasive tumor capsular or patients with direct tumor involvement with completely resected surrounding structures (pericardium, phrenic nerves, or lungs) were regarded as pathological R0 resection. Two patients with thymic and mediastinal fat invasion were regarded as R1 resection. We suppose that patients with capsular injury could be diagnosed with microscopic invasion of the tumor capsule on final histopathologic examination, and because of this, all patients presented postoperatively at multidisciplinary pathologic meetings to determine the indications for follow-up, radiotherapy, or chemotherapy, according to international guidelines [18]. Thus, we recommended adding radiotherapy only in the cases of microscopically invasive tumor capsular and thymic or mediastinal fat invasion.

The overall survival and recurrence rates reported for RATS and standard median sternotomy are similar, with high R0 resection rates of 90–94% for RATS [12,19]. Complete resection has been known to be a major prognostic factor for survival for thymectomy and is not negatively affected by the RATS approach [20].

The main outcome of our study is the length of hospital stay, which was significantly shorter in the RATS compared to the median sternotomy group (mean 3.64 vs. 10.67 days, *p* = 0.040). There are several limitations to the present study. First, the conclusions from this study are based on retrospective data because it is impossible to perform prospective comparative studies between RATS and median sternotomy approaches due to rapid advances in robotic-assisted thoracic surgery techniques. Second, the short follow-up period for cancer patients and patients with MG did not enable us to assess the long-term results of the RATS approach. Third, there is a case number discrepancy between the RATS and median sternotomy groups, both in sample size and baseline age. Patients undergoing sternotomy were substantially older, and age is independently associated with prolonged postoperative recovery and longer hospital stay. Although multivariable adjustment was applied, residual confounding cannot be excluded, particularly given the small size of the open surgery group. Consequently, the observed difference in hospital length of stay should be interpreted as an association rather than a direct effect of surgical approach. It is plausible that part of the longer hospitalization observed in the sternotomy group reflects age-related recovery differences rather than the surgical technique itself. We believe that, despite the limitations presented, with standardization, including proper patient selection, scrupulous surgical technique, and a longer follow-up period, additional advantages will be discovered.

## 5. Conclusions

In conclusion, while median sternotomy traditionally has been considered the standard approach for anterior mediastinal tumors, the evidence suggests that minimally invasive approaches, particularly RATS, can achieve comparable oncologic outcomes with potential perioperative benefits for appropriately selected patients. However, surgeon experience and careful patient selection remain important factors when considering RATS.

## Figures and Tables

**Table 1:** Clinical and surgical characteristics of the patients.

Variable	N	%	Notes	M	SD
Sex					
Male	54	48.6			
Female	57	51.4			
Age				57.19	23.10
Anterior mediastinal tumor surgery	111	100			
Type of operation					
RATS	93	83.8			
Median sternotomy	15	13.5			
Other approaches	3	2.7	Two VATS, one thoracotomy		
Side of RATS/VATS/thoracotomy					
Right	31	27.9	Thirty RATS, one VATS		
Left	65	58.6	Sixty-three RATS, one VATS, one thoracotomy		
Overall complications	11	9.9			
Atrial Fibrillation	3	2.7			
Empyema	2	1.8			
Phrenic nerve palsy *	2	1.8			
Mechanical ventilation	1	0.9			
Hemothorax	1	0.9			
Intraoperative bleeding	1	0.9			
Recurrent laryngeal nerve paresis	1	0.9			
Pneumonia	1	0.9			
Tracheostomy	1	0.9			
R0 resection for thymomas	63	56.8			
R0 (direct pericardial involvement)	7	6.3			
R0 (capsular invasion)	7	6.3	One with pericardial involvement		
R0 (lung and phrenic nerve involvement)	4 (2 lung and 2 phrenic nerve)	3.6	One converted to sternotomy, and one was performed initially by sternotomy		
R1 resection (thymic and mediastinal fat invasion)	2	1.8	One with pericardial involvement and both with capsular invasion		
M1 (pleural and diaphragm metastases)	1	0.9	With pericardial involvement		
Hospital Stay				4.62	6.04

* In two patients, the phrenic nerve was injured accidentally during dissection.

**Table 2:** Histopathologic characteristics of the patients *.

Tumor Pathology	111 pts, Mean Tumor Size *	MG, 38 pts	RATS, 93 pts *	Sternotomy, 15 pts	Other ***
Thymoma	65 pts (6.9 cm)	17 pts	51 pts ** (6.3 cm)	11 pts (9.1 cm)	3 pts
Thymus hyperplasia	27 pts	20 pts	27 pts		
Thymic cyst	3 pts	1 pt	3 pts		
Bronchogenic cyst	5 pts (3.2 cm)		4 pts (1.5 cm)	1 pt (9.1 cm)	
Thymic carcinoma	2 pts (3.8 cm)		2 pts (3.8 cm)		
Retrosternal goiter	1 pt (8.7 cm)			1 pt (8.7 cm)	
Lymphoma	1 pt (7.5 cm)			1 pt (7.5 cm)	
Paraganglioma	1 pt (6.1 cm)		1 pt (6.1 cm)		
Carcinoid	1 pt (3.7 cm)		1 pt (3.7 cm)		
Teratoma	1 pt (9.0 cm)			1 pt (9.0 cm)	
Cavernous hemangioma	1 pt (1.0 cm)		1 pt (1.0 cm)		
Other thymic benign pathology ****	3 pts		3 pts		

* For each pathology with a measurable tumor, its mean size is indicated in brackets; ** 1 pt from 51, after robotic thymectomy with no tumor on histopathologic examination, underwent sternotomy; *** 3 pts, 2 of them underwent VATS (6.5 cm) and 1 thoracotomy (9.0 cm); and **** 3 pts, with other benign thymic pathology (mediastinal cyst-2, mediastinal pseudocyst-1).

**Table 3:** Comparison between RATS vs. median sternotomy.

Variable	Median Sternotomy (n = 15)		RATS (n = 93)		χ^2^	t	*p*
	N (%)	M (SD)	N (%)	M (SD)			
Sex					0.46		0.49
Male	9 (60.0%)		47 (50.5%)				
Female	6 (40.0%)		46 (49.5%)				
Age		73.07 (31.11)		53.37 (19.95)		2.35	0.002
Anterior mediastinal tumor surgery	15 (100.0%)		75 (80.6%)		3.48		0.070
Chronic Diseases	10 (66.7%)		59 (63.4%)		0.06		0.809
Overall complications	1 (6.7%)		9 (9.7%)		0.14		0.709
Hospital Stay		10.67 (14.39)		3.64 (2.09)		1.88	0.040

**Table 4:** Logistic regression predicting overall complications.

Variable	OR	*p*	95% CI of OR	
			Lower bound	Upper bound
Sex (Male vs. Female)	1.78	0.50	0.34	9.31
Age	1.02	0.38	0.98	1.07
Anterior mediastinal tumor surgery	1.48	0.75	0.14	15.78
Chronic Diseases	0.26	0.13	0.05	1.49
Type of operation (RATS vs. median sternotomy)	2.19	0.56	0.15	31.10

**Table 5:** Linear regression for predicting hospital stay.

Variable	B	*p*	95% CI of B	
			Lower bound	Upper bound
Sex (Male vs. Female)	−1.73	0.20	−4.41	0.95
Age	0.03	0.35	−0.04	0.10
Anterior mediastinal tumor surgery	−0.10	0.96	−3.56	3.37
Chronic Diseases	−0.46	0.75	−3.30	2.37
Type of operation (RATS vs. median sternotomy)	−6.07	<0.001	−9.78	−2.36

## Data Availability

The data that support the findings of this study are available from the authors.

## Abbreviations

CO_2_	Carbon Dioxide CT-Computed Tomography
ECG	Electrocardiogram
HITHOC	Hyperthermic Intrathoracic Chemotherapy
LIA	Local Infiltration Anesthesia
MG	Myasthenia Gravis
MRI	Magnetic Resonance Imaging
MGFA	Myasthenia Gravis Foundation of America
OR	Odds Ratio
PFT	Pulmonary Function Tests
PVB	Paravertebral Block
RATS	Robotic-Assisted Thoracic Surgery
TEA	Thoracic Epidural Anesthesia
VATS	Video-Assisted Thoracic Surgery
